# pCysMod: Prediction of Multiple Cysteine Modifications Based on Deep Learning Framework

**DOI:** 10.3389/fcell.2021.617366

**Published:** 2021-02-23

**Authors:** Shihua Li, Kai Yu, Guandi Wu, Qingfeng Zhang, Panqin Wang, Jian Zheng, Ze-Xian Liu, Jichao Wang, Xinjiao Gao, Han Cheng

**Affiliations:** ^1^State Key Laboratory of Oncology in South China, Collaborative Innovation Center for Cancer Medicine, Sun Yat-sen University Cancer Center, Guangzhou, China; ^2^School of Life Sciences, Zhengzhou University, Zhengzhou, China; ^3^CAS Key Lab of Biobased Materials, Qingdao Institute of Bioenergy and Bioprocess Technology, Chinese Academy of Sciences, Qingdao, China; ^4^MOE Key Laboratory for Membraneless Organelles and Cellular Dynamics, Hefei National Laboratory for Physical Sciences at the Microscale, University of Science and Technology of China, Hefei, China

**Keywords:** protein cysteine modifications, feature extraction, deep learning, post-translational modifications, prediction

## Abstract

Thiol groups on cysteines can undergo multiple post-translational modifications (PTMs), acting as a molecular switch to maintain redox homeostasis and regulating a series of cell signaling transductions. Identification of sophistical protein cysteine modifications is crucial for dissecting its underlying regulatory mechanism. Instead of a time-consuming and labor-intensive experimental method, various computational methods have attracted intense research interest due to their convenience and low cost. Here, we developed the first comprehensive deep learning based tool pCysMod for multiple protein cysteine modification prediction, including *S*-nitrosylation, *S*-palmitoylation, *S*-sulfenylation, *S*-sulfhydration, and *S*-sulfinylation. Experimentally verified cysteine sites curated from literature and sites collected by other databases and predicting tools were integrated as benchmark dataset. Several protein sequence features were extracted and united into a deep learning model, and the hyperparameters were optimized by particle swarm optimization algorithms. Cross-validations indicated our model showed excellent robustness and outperformed existing tools, which was able to achieve an average AUC of 0.793, 0.807, 0.796, 0.793, and 0.876 for *S*-nitrosylation, *S*-palmitoylation, *S*-sulfenylation, *S*-sulfhydration, and *S*-sulfinylation, demonstrating pCysMod was stable and suitable for protein cysteine modification prediction. Besides, we constructed a comprehensive protein cysteine modification prediction web server based on this model to benefit the researches finding the potential modification sites of their interested proteins, which could be accessed at http://pcysmod.omicsbio.info. This work will undoubtedly greatly promote the study of protein cysteine modification and contribute to clarifying the biological regulation mechanisms of cysteine modification within and among the cells.

## Introduction

Post-translational modifications (PTMs) occur at specific amino acids extending the chemical repertoire of the 20 standard amino acids, which reversibly coordinate the signaling networks (Mann and Jensen, 2003; Mertins et al., 2013; Strzyz, 2016). Although cysteine (Cys) appears the least frequently among these common amino acids, it tends to act as a powerful molecular switch to maintain redox homeostasis and regulate a series of cell signaling transductions by PTMs (Marino and Gladyshev, 2011). The susceptibility of Cys to a variety of oxidative post-translational modifications is mainly dependent on the thiol groups, which are considerably more easily oxidized and highly nucleophilic (Brandes et al., 2009; Kumsta et al., 2011). According to different molecular conjugations to the thiol groups, cysteine modification can be classified into different types. Nitric oxide (NO) binding to some cysteine resides causes *S*-nitrosylation (Jia J. et al., 2014) and hydrogen sulfide (H2S) causes *S*-sulfhydration (Mishanina et al., 2015; Yang et al., 2015). Cumulated H_2_O_2_ reacting with cysteine leads to *S*-sulfenylation (Yang et al., 2015), *S*-sulfinylation (Akter et al., 2018), and *S*-sulfonylation (Lim et al., 2008). Cysteines can also bind metals such as Cu, Zn, and Fe to form iron-sulfur clusters and zinc finger domains (Oteiza, 2012; Rouault, 2015). The thioesterification reaction happened on lipid including *S*-prenylation and *S*-palmitoylation (Roth et al., 2006). These modifications lead to a cascade of biochemical reactions and regulate various physiological and pathological processes, such as autophagy (Carroll et al., 2018), protein stabilization (Kröncke and Klotz, 2009), redox homeostasis (Fra et al., 2017), and cell signaling (Hourihan et al., 2016), demonstrating a close relationship with many human diseases including cancers, diabetes, and so on. In this regard, to dissect the molecular mechanisms and regulatory roles of cysteine modification, it is urgently needed to precisely parse the potential cysteine modification sites and types.

With the rapid development of high-throughput sequencing and excellent specific chemical probes, cysteine modification profiles get unprecedented accumulation. For example, through a low-PH quantitation method, Fu et al. (2019) detected 1,547 sulfhydration sites on 994 proteins. Akter et al. (2018) identified and quantified 387 *S*-sulfinylation sites on 296 proteins in A549 and Hela cells. Recently, with label-free quantification strategy, Shen et al. (2017) identified 2,190 *S*-palmitoylated peptides on 883 proteins in liver. However, because the experimental methods are time consuming and labor intensive, the cysteine modification profiles expanded slowly, which significantly restricted the research on dissecting the molecular functions of cysteine modification. It is necessary to develop *in silico* tools to accurately predict cysteine modification sites, which will definitely promote the experimental identification of sophistical protein cysteine modification sites and types.

There are several computational tools used for predicting distinct cysteine modification types. For *S*-nitrosylation, Xue et al. (2010) collected 504 modification sites and constructed the first tool GPS-SNO for predicting *S*-nitrosylation sites. SNOSite (Lee et al., 2011b) predicted *S*-nitrosylation sites based on 586 experimental sites using support vector machine (SVM). iSNO-ANBPB (Jia C. et al., 2014) mainly adopted an adapted normal distribution bi-profile Bayes (ANBPB) feature extraction model. PreSNO (Hasan et al., 2019) used the LR model to integrate four encoding schemes with support vector machines and RF algorithms to predict SNO sites. In 2018, Xie et al. (2018) developed DeepNitro for the prediction of protein nitration and nitrosylation sites based on deep learning. iSulf-Cys (Xu et al., 2016) is the first program designed for predicting *S*-sulfenylation sites based on 1,105 sites quantified in RKO cells. Ju and Wang (2018) improved the model performance and developed Sulf_FSVM. MDD-Palm (Weng et al., 2017) can identify *S*-palmitoylation sites based on SVM. Recently, Ning et al. (2020) developed GPS-Palm using a deep learning based graphic presentation system for the prediction of *S*-palmitoylation. Although numerous predictors with considerable performance have been developed, the limitations are that all of these tools can predict just one kind of modification type and there is still room for improvement in model performance, while some modification types such as *S*-sulfinylation and *S*-sulfhydration are still lacking excellent predictors.

Previously, we have developed several protein post-translational modification tools for enzyme-specific lysine acetylation (Yu et al., 2020), calpain-specific cleavage site (Liu et al., 2019), and *S*-glutathionylation site (Li et al., 2020) prediction based on deep learning framework and particle swarm optimization (PSO) algorithm, which achieved significantly better performance than exiting tools. Traditional machine learning based method requires careful feature selection and scaling, which limited its performance. However, as a branch of machine learning, deep learning based method can fit high-dimensional features and clarify biological problems better than other algorithms. For example, Xu et al. (2017) constructed a predicting system for histone modification and discovered a potential embryonic stem cell (ESC) fate decision mechanism. DeepBind (Hassanzadeh and Wang, 2016) provided many candidate DNA-binding proteins by predicting DNA and protein-binding events. These results suggested an unprecedented excellent chance to utilize deep learning to solve biological problems. However, a credible deep learning framework is still lacking for comprehensive cysteine modification prediction.

In this work, after integrating the experimentally verified cysteine sites curated from literature and sites collected by other databases and predicting tools, we developed the first comprehensive deep learning based tool pCysMod for multiple protein cysteine modification prediction, including *S*-nitrosylation, *S*-palmitoylation, *S*-sulfenylation, *S*-sulfhydration, and *S*-sulfinylation. Seven sequence features including binary encoding profiles (BE), amino acid composition (AAC), position-specific scoring matrix (PSSM), and composition of k-spaced amino acid pairs (CKSAAP) were used to represent the sequences. These features were extracted and united into a deep learning model, and the hyperparameters were optimized by particle swarm optimization algorithms. Cross-validations indicated our model showed excellent robustness and outperformed existing tools. Besides, we constructed a comprehensive protein cysteine modification prediction web server based on this model to benefit the researches finding the potential modification sites of their interested proteins, which could be accessed at http://pcysmod.omicsbio.info.

## Methods

### Benchmark Dataset Preparation

The cysteine modification sites were collected in two major aspects. On the one hand, we curated the experimentally verified sites by searching the literatures from PubMed. For each modification, we used “nitrosylation,” “palmitoylation,” “sulfenylation,” “sulfhydration,” and “sulfinylation,” together with “cysteine” as our keywords. After traversing all related literatures in PubMed, we manually collected all experimentally verified sites. One the other hand, several databases and predictors with known cysteine modification sites were integrated, including GPS-SNO training dataset (Xue et al., 2010), Deep-Nitro training dataset (Xie et al., 2018), SNOSite training dataset (Lee et al., 2011b), GPS-Palm training dataset (Ning et al., 2020), iSulf-Cys training dataset (Xu et al., 2016), Sulf_FSVM training dataset (Ju and Wang, 2018), and dbPTM database (Huang et al., 2018). Finally, we obtained 23,041 *S*-nitrosylation sites in 10,671 proteins, 2,766 *S*-palmitoylation sites in 1,413 proteins, 4,978 *S*-sulfenylation sites in 3,288 proteins, 2,721 *S*-sulfhydration sites in 1,707 proteins, and 742 *S*-sulfinylation sites in 538 proteins as our final training dataset (Table 1 and Supplementary Table S1).

**TABLE 1:** A summary of each type of modification data.

Dataset	Human	Mouse	Rat	Other	Total
Number of *S*-nitrosylation sites (positive data)	10,784	4,103	1,629	2,819	38,670
Number of non-*S*-nitrosylation sites (negative data)	19,335				
Number of *S*-palmitoylation sites (positive data)	748	1,773	74	174	5,532
Number of non-*S*-palmitoylation sites (negative data)	2,766				
Number of *S*-sulfenylation sites (positive data)	2,587	352	1	1,806	9,492
Number of non-*S*-sulfenylation sites (negative data)	4,746				
Number of *S*-sulfhydration sites (positive data)	2,010	0	0	525	5,070
Number of non-*S*-sulfhydration sites (negative data)	2,535				
Number of *S*-sulfinylation sites (positive data)	440	0	208	7	1,310
Number of non-*S*-sulfinylation sites (negative data)	655				

To generate the positive and negative datasets, we retrieved the protein sequence from UniProt database (UniProt Consortium [UC], 2015) for each protein. For each modification, the golden positive dataset was the modification sites from the benchmark dataset, whereas all cysteine sites that were not modified on the same protein were treated as the negative dataset. The sequence box for feature extraction consists of a cysteine in the middle and 15 upstream and downstream amino acids at both ends. For the peptide of less than 31-amino acids, pseudo-amino acids “^∗^” were added to make sure the peptides were of equal length. If the sequence in the negative dataset was the same as the positive set in the same cysteine modification, only the sequence in the positive data set is preserved. In addition, due to the high imbalance between positive and negative samples, we randomly selected the same number of negative samples to ensure that the number of positive peptides was equal to the number of negative peptides (Zhao et al., 2012). At the same time, we used CD-Hit (Fu et al., 2012) with a threshold of 90, 80, and 70% sequence similarity treatment on a short peptide consisting of 31-amino acids, and then performed fivefold cross-validation. In this work, cross-validations were used to evaluate the performance of the model. Since cross-validation is an efficient way of examining the robustness and accuracy of a predicting model, it is unnecessary to divide the benchmark dataset into training set and testing set (Zhang et al., 2020).

### Feature Extraction

#### Binary Encoding Profiles

Binary encoding (BE) (Song et al., 2010) was derived from computational programming, which uses the binary digit, that is, “0” or “1,” as the fundamental unit of information. Each printable character can be uniquely represented by combining bits. As mentioned above, each peptide in the benchmark dataset consists of at most 21 types of amino acids, which are ACDEFGHIKLMNPQRSTVWY^∗^. Hence, a 21-dimentional binary vector was used to represent each amino acid. For example, “A” was encoded as (100000000000000000000), “E” was encoded as (000100000000000000000), and the pseudo-amino acid “^∗^” was encoded as (000000000000000000001). In this regard, each peptide was represented by a 651-dimensional vector.

#### Amino Acid Composition

The amino acid composition (AAC) is an important feature to identify β-barrel membrane proteins (Radivojac et al., 2010; Lee et al., 2011a), which stand for the occurrence frequency of 21-amino acids on any specific peptides. The feature length of this encoding method is 21 for each peptide.

#### Position-Specific Scoring Matrix

Position-Specific Scoring Matrix (PSSM) was first introduced as an alternative to consensus sequences (Stormo et al., 1982); this feature was derived from a set of functionally related aligned sequences, which is commonly used for computational motif discovery in biological sequences (Stormo, 2000). For a group of given peptides, PSSMs assume that the probabilities for each position are statistically independent and calculate the probability for each specific amino acid at a particular position. The probabilities for a particular position sum up to 1. In this work, we calculated PSSMs for positive dataset and negative dataset, so the dimension of this feature is 62.

#### Composition of *k*-Spaced Amino Acid Pairs

The encoding scheme based on the Composition of *k*-Spaced Amino Acid Pairs (CKSAAP) (Zhao et al., 2012) is an effective feature extraction method, which can reflect the information of amino acid pair motifs in a set of peptides. The *k*-spaced means two amino acids in a peptide separated by k-amino acids, and CKSAAP encoding calculates the occurrence frequency for each pair. When *k* = 0, it means the occurrence frequency of each pair is composed of adjacent amino acids, and the dimension is 441. In this work, after taking computation and time cost into consideration, we merely adopted *k* = 0, 1, 2, and 3, and the final dimension of this method is 1,764.

### Model Construction

Although each modification type has a special benchmark dataset and needs a special model to fit, they have analogous model architectures. Here, we introduce a general deep learning based model architecture used in this work for cysteine prediction. For each modification type, the benchmark peptide dataset was encoded by four feature extraction methods mentioned above. The model received the numerical transferred sequences in the input layer, which consists of four independent DNN submodules to train four input features. Then the four submodules were merged and flattened into a fully connected layer after sufficiently learning the features. Finally, pCysMod output a probability of whether this peptide could undergo particular modification. Early stopping and dropout functions were used to make sure the training set was not over-represented. To optimize the numerous hyperparameters in pCysMod, particle swarm optimization algorithm was applied to generate the maximum performance as previously reported (Yu et al., 2020). The python package “pyswarm”^1^ as used.

### Performance Evaluation

Four common measurements were adopted to evaluate the performance of pCysMod as previously described (Liu et al., 2012), including specificity (Sp), sensitivity (Sn), accuracy (Ac), and Mathews correlation coefficient (MCC). The detailed descriptions of these four measurements are defined as below:

(1) $$\mathrm{Sn}=\frac{\text{TP}}{\mathrm{TP}+\mathrm{FN}}$$

(2) $$\mathrm{Sp}=\frac{\text{TN}}{\mathrm{TN}+\mathrm{FP}}$$

(3) $$\text{Ac}=\frac{\mathrm{TP}+\mathrm{TN}}{\mathrm{TP}+\mathrm{FP}+\mathrm{TN}+\mathrm{FN}}$$

(4) $$\text{MCC}=\frac{{\mathrm{TP}}^{*}\,\mathrm{TN}-{\mathrm{FP}}^{*}\,\mathrm{FN}}{\sqrt{\left(\mathrm{TP}+\mathrm{FP}\right)\,\left(\mathrm{TP}+\mathrm{FN}\right)\,\left(\mathrm{TN}+\mathrm{FP}\right)\,\left(\mathrm{TN}+\mathrm{FN}\right)}}$$

We calculated the area under the receiver operating characteristic (ROC) curve (AUC) values to show the model performance. Four-, six-, eight-, and tenfold cross-validations were used to evaluate the robustness and accuracy of pCysMod. Tenfold cross-validation was used to compare the performance of pCysMod with the existing tools.

### Implement of the Web Server

pCysMod model was constructed by Keras, with TensorFlow as its backend implementation. The secondary structure and surface accessibility information of the query sequence were calculated by NetSurfP (Petersen et al., 2009), and the disorder information was predicted by IUPred (Dosztanyi et al., 2005). The web server was built in PHP and Python, which could be accessed at http://pcysmod.omicsbio.info.

## Results

### The Construction of Computational Model to Predict Cysteine Modification Sites

**FIGURE 1:**
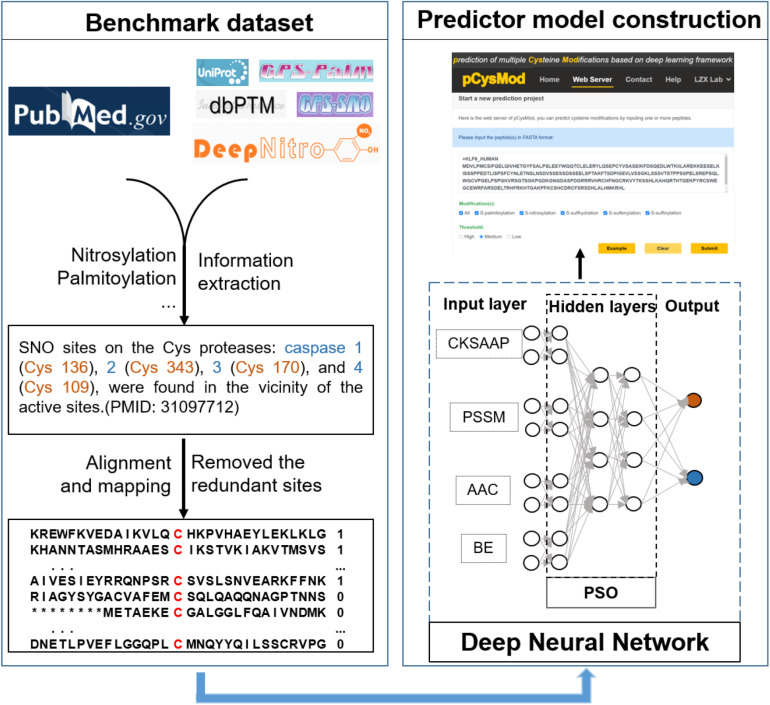
An overview of the model.

Cysteine modification sites were obtained in the literature and other predictive tools (Figure 1). After removing redundant sequences and balancing the datasets, we finally obtained 19,335 *S*-nitrosylation-positive sites, 2,766 *S*-palmitoylation-positive sites, 4,746 *S*-sulfenylation-positive sites, 2,535 *S*-sulfhydration-positive sites, and 655 *S*-sulfinylation-positive sites. The number of negative and positive sequences of different modifications was the same and shown in Table 1. Then, we developed the first model to predict multiple cysteine modifications named pCysMod. The software was based on deep learning and PSO algorithm. The sequence features were extracted by four methods, including BE, AAC, PSSM, and CKSAAP (Figure 1). Furthermore, we used Python, PHP, JavaScript, and HTML to construct pCysMod online server, which can be accessed through http://pcysmod.omicsbio.info.

### The Characteristic of Cysteine Modification Sites and Proteins

**FIGURE 2:**
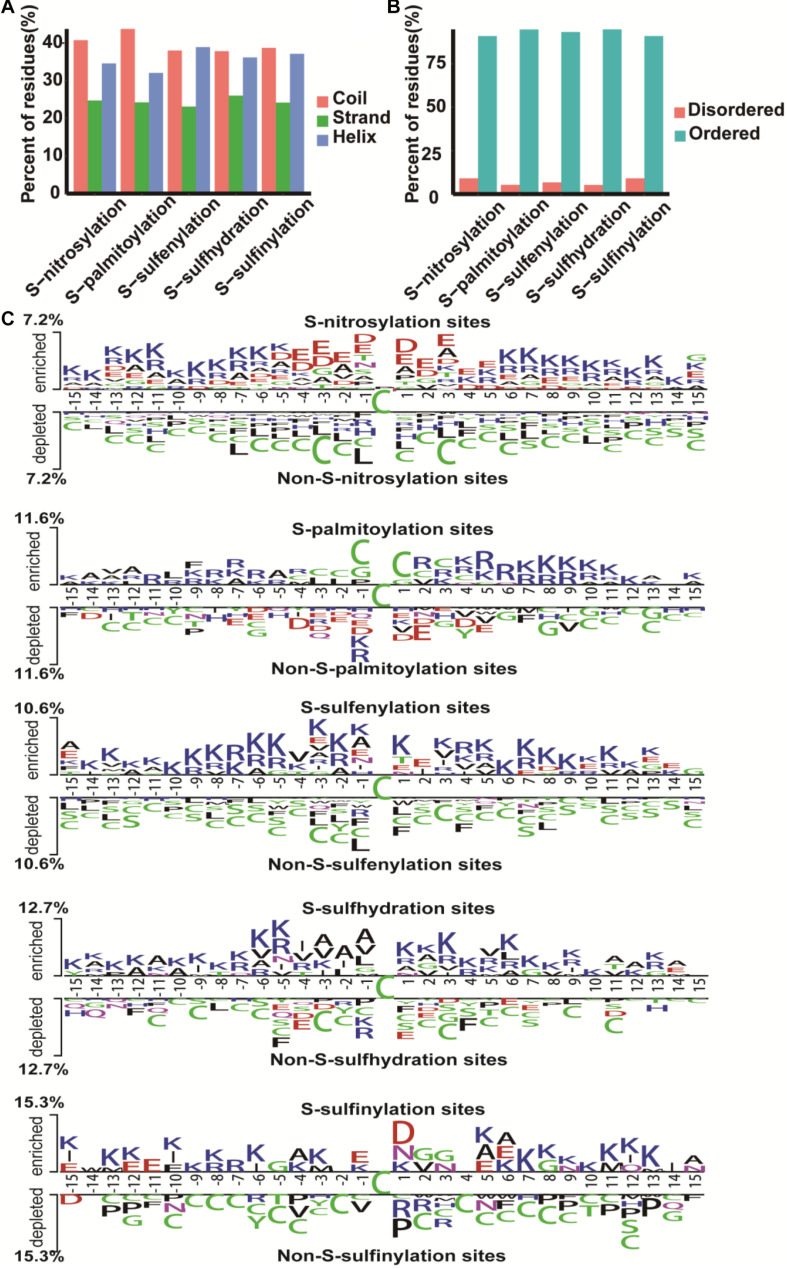
The characteristic of cysteine modification sites and proteins. **(A)** The secondary structure. **(B)** The disorder information of cysteine modification sites. **(C)** Preference for amino acids around the cysteine modification sites and non-cysteine modification sites.

To better understand the structure of different cysteine modification sites, we used the secondary structure prediction algorithms PsiPred (McGuffin et al., 2000) and IUPred (Dosztanyi et al., 2005) to classify the cysteine sites of all proteins. The *S*-nitrosylation sites and *S*-palmitoylation sites were predominantly distributed in coil, while *S*-sulfenylation, *S*-sulfhydration, and *S*-sulfinylation sites in coil and helix were relatively close (Figure 2A), and the cysteine sites were mainly predicted to locate in ordered regions (Figure 2B). Furthermore, we used Two Sample Logo (Vacic et al., 2006) to analyze amino acid preference. The difference between *S*-sulfinylation sites and non-*S*-sulfinylation sites are shown in Figure 2C. Lysine and asparagine residues were enriched around the *S*-sulfinylation sites, but cysteine residues were deleterious to the modification. In *S*-nitrosylation cysteine modification, the asparagine and glutamic were enriched near the modification site (Figure 2C). Lysine residues also tended to be *S*-sulfenylated and *S*-sulfhydrated, while cysteine residues were enriched in *S*-palmitoylation cysteine modification (Figure 2C).

Using the collected human proteins with different cysteine modifications, we conducted GO and KEGG enrichment by clusterProfiler (Yu et al., 2012). We found that the mostly enriched biological processes were catabolic process in *S*-sulfinylation and *S*-nitrosylation, such as carboxylic acid catabolic process and organic acid catabolic process (Supplementary Figure S1). *S*-Sulfenylation and *S*-sulfhydration were related to transcription, and *S*-palmitoylation tended to affect transduction (Supplementary Figure S1). Based on the enrichment results of GO cellular components, we observed that ribosome was enriched in different cysteine modifications (Supplementary Figure S1). GO molecular function and KEGG pathway analyses also indicated that the cysteine modifications other than *S*-palmitoylation were involved in the redox process (Supplementary Figures S1, S2). The results were consistent with previous studies, which showed that *S*-nitrosylation, *S*-sulfenylation, *S*-sulfhydration, and *S*-sulfinylation play critical roles in oxidative post-translational modifications (Chung et al., 2013).

### Evaluating the Performance of pCysMod

**FIGURE 3:**
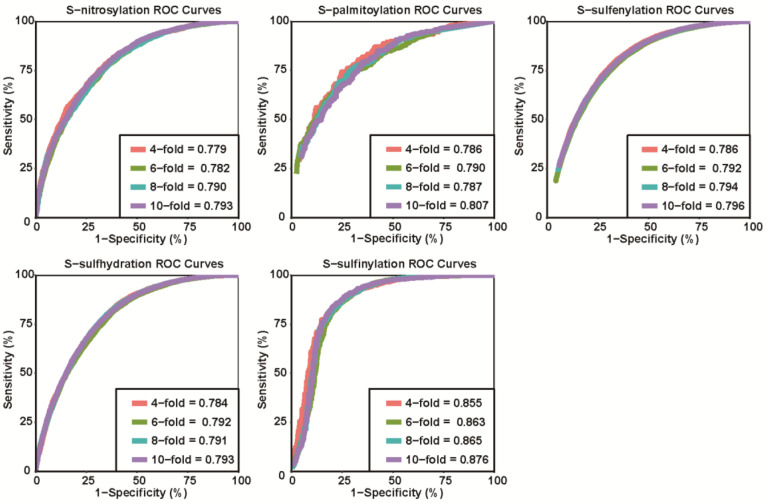
The ROC curves and AUCs of 4-, 6-, 8-, and tenfold cross-validations are shown.

We generated the first model to predict multiple types of cysteine modification based on the method mentioned above. Four-, six-, eight-, and tenfold cross-validations were used to evaluate the accuracy and robustness of pCysMod. The ROC curves and AUC values are displayed in Figure 3. The best cross-validation AUC values for *S*-nitrosylation, *S*-palmitoylation, *S*-sulfenylation, *S*-sulfhydration, and *S*-sulfinylation were 0.793, 0.807, 0.796, 0.793, and 0.876. The similar and considerable performance declared the robustness and high accuracy of pCysMod. Since cross-validation is an efficient way of examining the robustness and accuracy of a predicting model, it is unnecessary to divide the benchmark dataset into training set and testing set (Zhang et al., 2020). We tested the predictive performance of different feature extractions. The fivefold cross-validation AUCs were calculated for different features, and the results are visualized in the added Supplementary Figure S3, which indicated that combining multiple features can obtain more stable prediction performances. Not only that, in order to avoid the overestimation of prediction performance due to the possible high similarity of the sequences, we used CD-Hit with a threshold of 70, 80, and 90% sequence similarity analysis on short peptides composed of 31-amino acids, and then performed fivefold cross-validation based on the clustering results. Compared with only removing redundant peptides, the results showed that not using CD-Hit did not lead to an overestimation of the prediction performance (Supplementary Table S2).

We then performed tenfold cross-validation to demonstrate the superiority of pCysMod compared with existing tools, including *S*-nitrosylation site-predicting tools GPS-SNO (Xue et al., 2010), Deep-Nitro (Xie et al., 2018), iSNO-ANBPB (Jia C. et al., 2014), and PreSNO (Hasan et al., 2019), *S*-palmitoylation site-predicting tools GPS-Palm (Ning et al., 2020) and MDD-Palm (Weng et al., 2017), and *S*-sulfenylation site-predicting tools iSulf-Cys (Xu et al., 2016) and Sulf_FSVM (Ju and Wang, 2018). The performances of these predictors were retrieved from previous reported literatures, which are shown in Table 2. Through the comparison, we can conclude that the performance of pCysMod is higher than or equal to existing predictors, showing a considerable predictive power for general cysteine modification prediction.

**TABLE 2:** Performance comparison of pCysMod with other predictors.

CysMod	Predictor	Sn (%)	Sp (%)	Ac (%)	MCC	AUC
*S*-Nitrosylation	GPS-SNO	53.57	80.14	75.80	0.286	0.524
	DeepNitro	40.0	85.0	77.7	0.236	0.743
	PreSNO	60.4	76.9	75.2	0.252	0.756
	iSNO-ANBPB			67.01	0.351	
	pCysMod	61.09	80.02	70.57	0.420	0.793
*S*-Palmitoylation	GPS-Palm	68.47	85.04	82.67	0.448	0.855
	MDD-Palm	74.0	74.0	74.0	0.40	0.80
	pCysMod	62.91	80.29	71.66	0.439	0.807
*S*-Sulfenylation	iSulf-Cys	67.31	63.89	65.59	0.312	0.715
	Sulf_FSVM	68.54	68.03	68.29	0.365	0.747
	pCysMod	75.66	70.08	72.84	0.458	0.796

Finally, we have constructed an independent predictor for each modification, with the same basic structure and distinct hyperparameters. At the same time, we tested the cross differentiating capabilities of five cysteine modification predictors, that is, using the constructed model to predict other types of cysteine modification. The prediction results show that, different predictors have specificity for their corresponding modification type (Supplementary Table S3). Although the basic structure of each modified model is the same, the internal parameters adjusted by the PSO algorithm are distinct, showing a different modification feature and pattern of each modification type.

### Implementation of pCysMod Web Server

**FIGURE 4:**
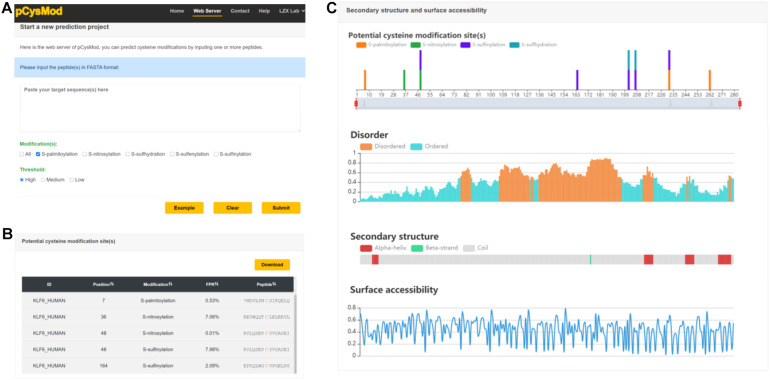
The web server of pCysMod. **(A)** The prediction page. **(B)** Potential cysteine modification sites. **(C)** Secondary structure and surface accessibility.

In order to provide an efficient and convenient way to facilitate basic research, we generated the first comprehensive cysteine modification prediction web server pCysMod. We tested the pCysMod website on various commonly used web browsers, such as Google Chrome, Internet Explorer, and Mozilla Firefox to provide a robust service. The prediction and results pages are shown in Figure 3. The input text box required FASTA format protein sequence, and then we should select which type of modification is needed to be predicted and its threshold (Figure 4A). The prediction information was organized by two aspects and displayed in the results page, including “Potential cysteine modification sites” (Figure 4B) and “Secondary structure and surface accessibility” (Figure 3C). The detailed modification sites and types information are displayed in the “Potential cysteine modification sites” section (Figure 4B), and the sequence structure properties such as disordered information, secondary structure, and surface accessibility features are shown in the “Secondary structure and surface accessibility” (Figure 4C). When multiple protein sequences were submitted, pCysMod will predict and show the first one as a default. By clicking the selection box, users can choose which protein to display, and this will take 20 s in average. Besides, the proteins and peptides used in this study were uploaded in the web server and users can download the relevant data in the “Help” section. Overall, pCysMod was the first comprehensive cysteine modification prediction web server, which will undoubtedly greatly promote the study of protein cysteine modification and contribute to clarifying the biological regulation mechanisms of cysteine modification within and among the cells.

## Discussion

Protein cysteine modifications lead to a series of biochemical reactions, regulate various physiological and pathological processes, such as autophagy (Carroll et al., 2018), protein stabilization (Kröncke and Klotz, 2009), redox homeostasis (Fra et al., 2017), and cell signaling (Hourihan et al., 2016), demonstrating a close relationship with many human diseases including cancers, diabetes, and so on. Although many efforts have been made in this field, the experimental identification of cysteine modification proteins is tedious and laborious and the underlying molecular mechanisms are still unclear. In this regard, to dissect the molecular mechanisms and regulatory roles of cysteine modification, it is urgently needed to precisely parse the potential cysteine modification sites and types.

Through carefully curated previous reported literatures, predictors, and databases, we generated a benchmark dataset that consists of five types of cysteine modification, including *S*-nitrosylation, *S*-palmitoylation, *S*-sulfenylation, *S*-sulfhydration, and *S*-sulfinylation. The cysteine modification sites prefer to enrich in ordered regions. Consistent with previous reports, *S*-nitrosylation, *S*-sulfenylation, *S*-sulfhydration, and *S*-sulfinylation play crucial roles in oxidative post-translational modifications (Chung et al., 2013). Besides, the thioesterification reaction can cause *S*-palmitoylation by reversibly adding one or multiple palmitoyl moieties to cysteine residues (Roth et al., 2006), and *S*-palmitoylation also mediates a series of biochemical reactions, such as metabolism (Shen et al., 2017) and autophagy (Kim et al., 2019).

Then, we generated the pCysMod to predict multiple types of cysteine modification. Four-, six-, eight-, and tenfold cross-validations declared the robustness and high accuracy of pCysMod. Tenfold cross-validation comparison indicated a considerable predictive power for general cysteine modification prediction. We further generated the first comprehensive cysteine modification prediction web server pCysMod to provide an efficient and convenient way to facilitate basic research.

Although pCysMod has performed excellently in predicting cysteine modification, the limitations still exit. Currently, the cysteine modification data are still limited. We will keep collecting more modification types for future plans to generate a more comprehensive cysteine modification predictor. Furthermore, more deep learning methods could be taken into consideration, such as graph convolutional neural network (GCN), capsule network, and attention mechanisms, which may be an important and meaningful approach to help improving the current performance.

## Data Availability Statement

The original contributions presented in the study are included in the article/Supplementary Material, further inquiries can be directed to the corresponding author/s.

## Author Contributions

JW, XG, and HC designed and supervised the experiments. SL, KY, and GW performed the experiments and data analysis, and developed the predictor. QZ and PW contributed to data analysis and predictor development. SL, KY, and GW wrote and revised the manuscript with contributions of all authors. All authors reviewed the manuscript.

## Conflict of Interest

The authors declare that the research was conducted in the absence of any commercial or financial relationships that could be construed as a potential conflict of interest.

## Footnotes

1 https://github.com/tisimst/pyswarm

## Abbreviations

PTMs: post-translational modifications
Cys: cysteine
H_2_S: Hydrogen sulfide
NO: nitric oxide
SVM: support vector machine
ESC: embryonic stem cell
PSO: particle swarm optimization
CKSAAP: composition of k-spaced amino acid pairs
BE: binary encoding profiles
PSSM: position-specific scoring matrix
AAC: amino acid composition
Sp: specificity
Sn: sensitivity
Ac: accuracy
ROC: receiver operating characteristic
AUC: area under ROC curve
MCC: Mathews correlation coefficient
DNN: deep neural network
GCN: graph convolutional neural network.
