# Supplementary figures and images for: pCysMod: Prediction of Multiple Cysteine Modifications Based on Deep Learning Framework
Source: Front Cell Dev Biol. 2021 Feb 23;9:617366. doi: 10.3389/fcell.2021.617366 (PMC7959776; doi:10.3389/fcell.2021.617366)

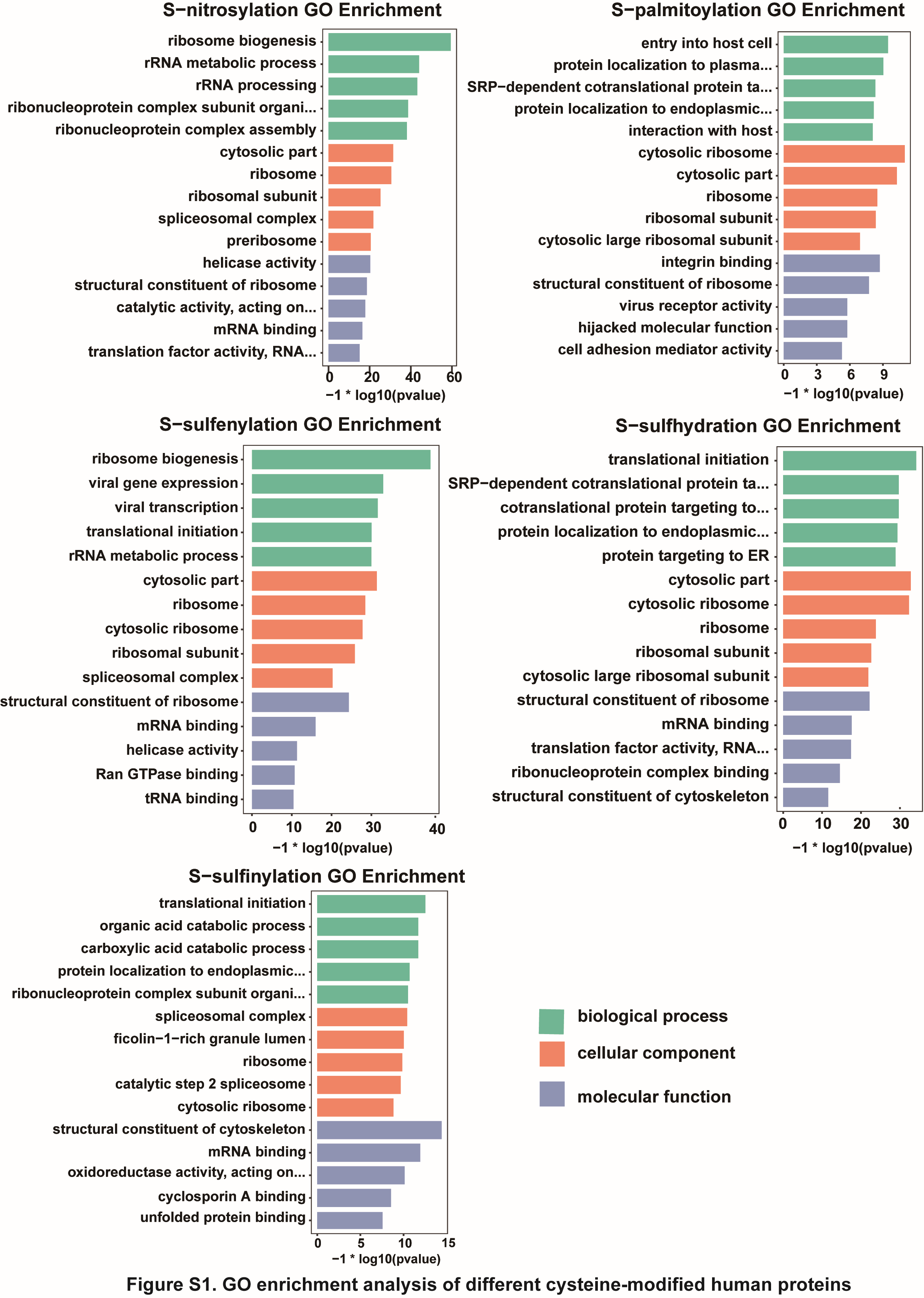

Supplement: Supplementary file 1 [file Image_1.TIF]

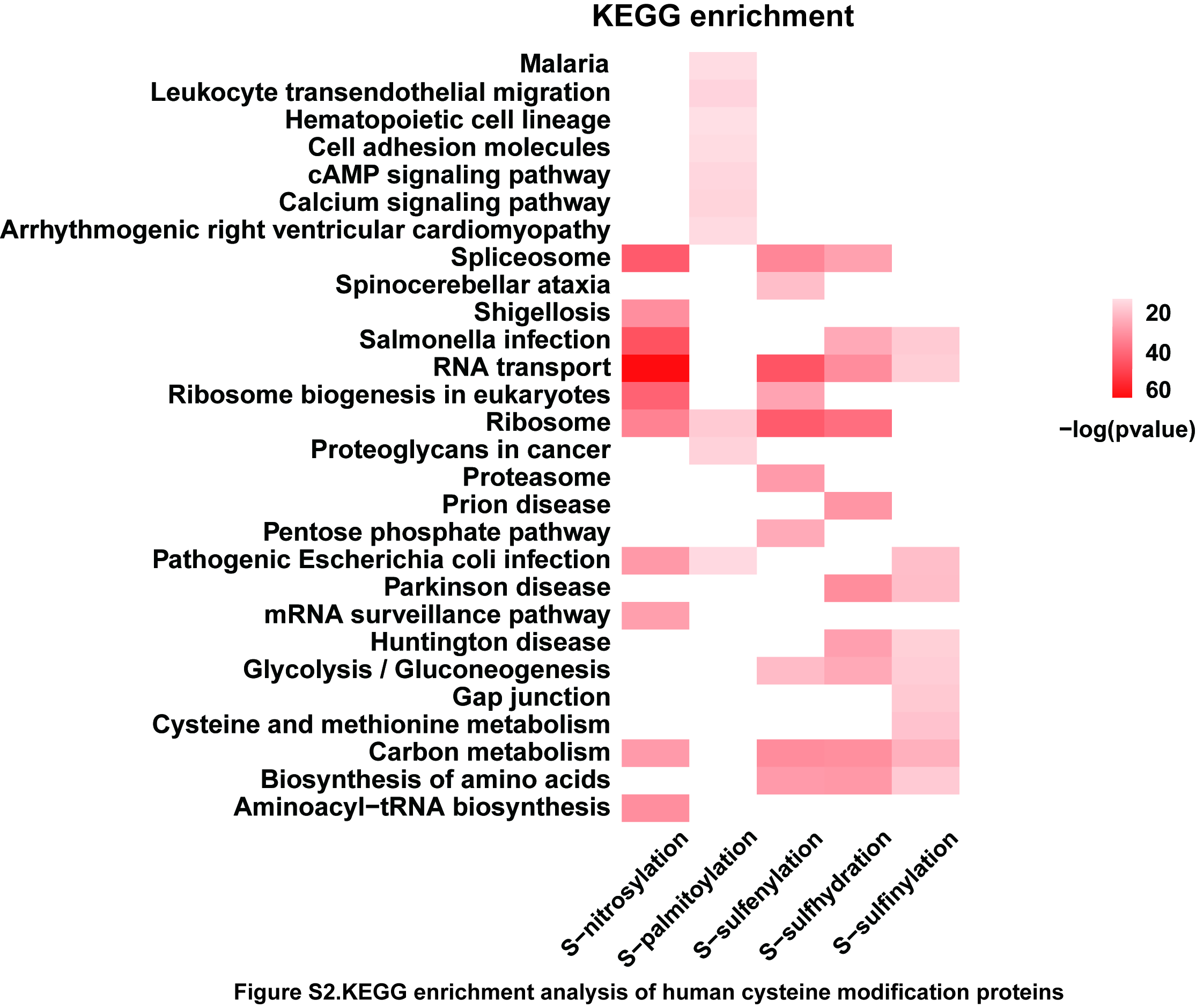

Supplement: Supplementary file 2 [file Image_2.TIF]

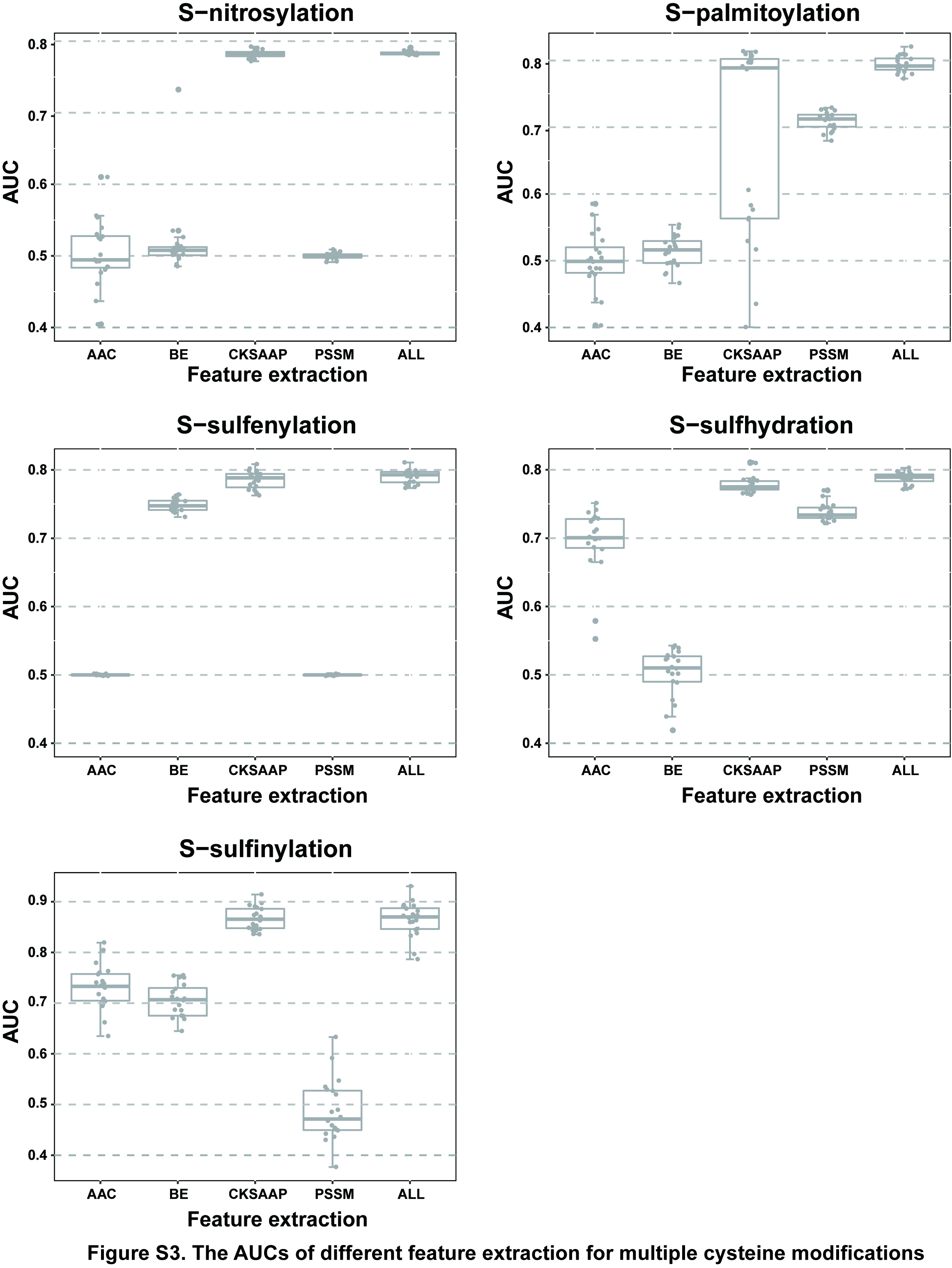

Supplement: Supplementary file 3 [file Image_3.TIF]
