# Supplementary material for: pCysMod: Prediction of Multiple Cysteine Modifications Based on Deep Learning Framework
Source: Front Cell Dev Biol. 2021 Feb 23;9:617366. doi: 10.3389/fcell.2021.617366 (PMC7959776; doi:10.3389/fcell.2021.617366)
Supplement: Supplementary file 7 [file Table_4.DOCX]

**Supporting Information**

**Figure S1.** GO enrichment analysis of different cysteine-modified human proteins.

**Figure S2.** KEGG enrichment analysis of human cysteine modification proteins.

**Figure S3.** The AUCs of different feature extraction for multiple cysteine modifications.

**Table S1.** Experimentally identified cysteine modification sites.

**Table S2.** The performance of 5-fold cross-validation after different processing methods.

**Table S3.** The cross-differentiating capabilities among the five cysteine modifications.
